# Project SoL—A Community-Based, Multi-Component Health Promotion Intervention to Improve Healthy Eating and Physical Activity Practices among Danish Families with Young Children Part 2: Evaluation

**DOI:** 10.3390/ijerph15071513

**Published:** 2018-07-18

**Authors:** Bent Egberg Mikkelsen, Paul Bloch, Helene Christine Reinbach, Tine Buch-Andersen, Lise Lawaetz Winkler, Ulla Toft, Charlotte Glümer, Bjarne Bruun Jensen, Jens Aagaard-Hansen

**Affiliations:** 1 Department of Learning and Philosophy, Aalborg University, Copenhagen DK-2450, Denmark; 2 Steno Diabetes Center Copenhagen, Health Promotion, Niels Steensens Vej 6, DK-2820 Gentofte, Denmark; paul.bloch@regionh.dk (P.B.); Bjarne.Bruun.Jensen@regionh.dk (B.B.J.); jens.aagaard-hansen@regionh.dk (J.A.-H.); 3 Department of Development and Planning, Aalborg University, Copenhagen DK-2450, Denmark; hcr@plan.aau.dk (H.C.R.); tba@plan.aau.dk (T.B.-A.); 4 Research Centre for Prevention and Health, Capital Region, Glostrup DK-2600, Denmark; Lawaetzlise@gmail.com (L.L.W.); ulla.toft@regionh.dk (U.T.); FS0H@suf.kk.dk (C.G.); 5 MRC Developmental Pathways for Health Research Unit, Faculty of Health Sciences, University of the Witwatersrand, Johannesburg ZA-2000, South Africa

**Keywords:** action research, children, healthy eating, community-based, multi-component interventions, complex interventions, cross-disciplinarity, realistic evaluation, settings

## Abstract

Project SoL is implemented over a period of four years with the aim to promote healthy eating and physical activity among children aged 3–8 years by targeting the families in a Danish municipality based on the multi-component, supersetting strategy. Interventions are implemented in childcare centres, schools and supermarkets in three local communities as well as in local mass media and social media during a 19 months period in the Municipality of Bornholm. The matching Municipality of Odsherred serves as a control site based on its similarity to Bornholm regarding several socio-demographic and health indicators. The present paper gives an account of the design used for the summative and formative evaluation based on a realistic evaluation and a mixed methods approach combining qualitative and quantitative methods. Summative studies are conducted on changes of health behaviours among the involved families and within the municipalities in general, changes in community awareness of the project, changes in purchase patterns, changes in overweight and obesity among the targeted children and changes in knowledge and preferences among children due to sensory education workshops. The formative research comprises studies on children’s perceptions of health, perceptions of staff at supermarkets and media professionals on their roles in supporting the health promotion agenda, and motivations and barriers of community stakeholders to engage in health promotion at community level. The paper discusses operational issues and lessons learnt related to studying complex community interventions, cross-disciplinarily, interfaces between practice and research and research capacity strengthening; and suggests areas for future research. The development and implementation of the intervention and its theoretical foundation is described in a separate paper.

## 1. Introduction

Interventions targeting individuals through educational approaches have shown limited effects in changing eating behavior and as a result more research has been initiated to study the potential impact of environmental approaches. Interventions in settings such as kindergarten [1,2], schools [3,4], worksites [5,6]) and food stores [7,8] have shown some effect. Project SoL—from the Danish “Sundhed og Lokalsamfund” (Health and Local Community) went a step further by combining an environmental approach targeting more levels and more settings in a community wide strategy. SoL is a research and development project aimed at promoting healthier lifestyles among Danish children aged 3 to 8 years and their families. Project SoL is a community-based, multi-component intervention project, which is implemented in multiple settings in a coordinated manner based on the supersetting approach with its emphasis on integrated actions, participation and empowerment of citizens, respect for local contexts and research generation and use [9]. The project is carried out in Denmark over a period from 2012–2015. The first part of the project includes a 19 months period during, which interventions are implemented in childcare centres, primary schools, supermarkets and local mass media, in three high-intensity local communities in the Municipality of Bornholm. The remaining part the island of Bornholm is only targeted through the media intervention components and can therefore be considered low-intensity communities. The planning and implementation of the intervention is described in detail in [9,10].

The aim of the present paper is to describe the evaluation design of Project SoL. Thus, the paper provides details of the overall study design as well as the objectives, designs and methods of the main quantitative and qualitative sub-studies conducted as part of the summative and formative evaluation of the project. Furthermore, the paper discusses the lessons learnt regarding evaluation of complex interventions, cross-disciplinarily, collaboration between researchers and practitioners, the additional role of researchers as practitioners and research capacity strengthening as encountered by the project.

## 2. Overall Research Methodology

### 2.1. Research Design

Complex interventions involve a multitude of participants and stakeholders and take place within open social systems unfolding in the many settings used by people in everyday life, they cannot be evaluated solely by using randomized, controlled trials. Consequently, the Project SoL evaluation methodology is based on two conceptual cornerstones, realistic evaluation and mixed methods. Realistic evaluation is one of the approaches that have been developed for the evaluation of this kind of interventions [11]. The project includes studies with both quantitative and qualitative methods in accordance with the mixed methods approach [12]. The summative studies rely primarily on quantitative behaviour change data serving as proxies for health outcome variables, whereas the formative evaluation of key processes relies primarily on qualitative methods. In addition, the project takes inspiration from the guidelines for the evaluation of complex interventions [13,14].

The theory-based realistic evaluation [11] has its roots in the implementation science [15]. Realistic evaluation is based on a programmed theory that describes how intervention components are believed to interact and provides a graphical representation of the expected cause-effect relationships of the project including its proximal and distal outcomes. It consists of three main components: (1) context (influence of the socio-cultural and political surroundings within which the intervention is implemented); (2) mechanisms (steps on the pathways leading to change and the relationship between those factors); and (3) outcomes (assumed proximal effects of the project activities). Based on this, nodes of context, mechanisms and outcomes (CMOs) are identified which become foci of sub-studies. The programmed theory guided both the summative and the formative evaluation studies of Project SoL.

The summative part of the research is designed as a quasi-experimental study which matched the Municipalities of Bornholm (intervention) and Odsherred (control) at two levels. The three local, high-intensity intervention communities at Bornholm are compared to three equivalent non-intervention communities in the Municipality of Odsherred, whereas other sub-studies compares samples of citizens in the two municipalities at large.

We use the term “evaluation” as a systematic assessment of a concrete intervention, and the term “research” as a more comprehensive endeavor to draw universal conclusions of relevance outside the field of study. The activities described in the present article comprise both aspects, and we use the terms interchangeably.

The project is organized as a formalized partnership between the three participating research institutions with Aalborg University (consortium leader) and Steno Diabetes Center Copenhagen and Research Centre for Prevention and Health as well as key local stakeholders in the municipalities and institutions in the three high-intensity communities. For more details on the organizational structure of Project SoL, see [9].

### 2.2. Research Ethics

Project SoL is approved by the Danish Data Protection Agency according to the Danish Act on Processing of Personal Data. A separate ethics approval is obtained from the Institutional Review Board, the Ethical Committee of the Capital Region (H-3-2013-036) for a study testing the validity and reproducibility of the semi-quantitative food-frequency questionnaire. Approval from the Danish Health Research Ethics Committee System is not required according to Danish regulations.

Data is extracted from the Danish CPR (social security number-register), which is a register containing a unique ID reference of all citizens and that allows for precise sampling of subjects for research purposes. The purpose is twofold: (1) To extract addresses of randomly selected adult citizens and; (2) To get information on socio-demographics and family status for the inclusion as covariates in the statistical analyses. No children are sampled through the CPR register.

All data is stored and treated with confidentiality. Data are published with due consideration to anonymity. Where relevant, the studies apply oral informed consent to participate in accordance with all stipulations of the Helsinki Declaration, including the purpose and content of the research, the right to decline and withdraw from the study as well as the risks and benefits of participation, the anonymity of study participants, the confidentiality of data handling and the contact details of researchers. Parental informed written consent is obtained for all children participating in the anthropometric sub-study and the validation study.

### 2.3. Research Capacity Building

Project SoL provided research capacity strengthening as a central element of the research activities. This includes two PhD students and two postdoc researchers who evaluated different parts of the intervention.

In addition, drawing on the principles of service learning [16] and knowledge triangles [17] several Master’s and Bachelor students conducts supplementary sub-studies (for a full list, see Table A1 in Appendix A). These students are co-supervised by their university supervisors and senior project researchers. Research seminars are held at regular intervals to ensure common understanding of research concepts and goals, and to enhance cohesion and synergy between the different research disciplines and sub-studies.

## 3. Summative Research

### 3.1. General Issues

**Figure 1:**
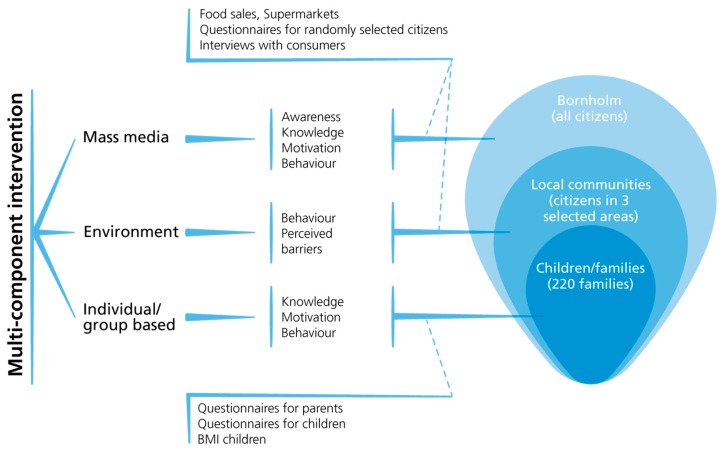
Conceptual model for the summative evaluation of the SoL intervention.

The programmed theory of Project SoL indicates how the intervention components are configured to address the core problems and causes and how are believed to lead to proximal and distal outcomes according to selected configurations of contexts, mechanisms and outcomes (CMOs). The summative evaluation assesses the effect of the SoL intervention on health behaviour focused on changes in dietary intake and level of physical activity (Figure 1). The same variables are measured in the intervention municipality (Bornholm) and the control municipality (Odsherred) based on questionnaire surveys before and after the intervention, and in some cases a mid-term assessment is also conducted during the intervention at Bornholm. An overview of the data collection activities is shown in Table 1.

### 3.2. Summative Studies

The overall summative evaluation comprises six sub-studies and focuses on change within various domains: health behaviours among the families in high-intensity local communities, health behaviours among citizens at municipality level, awareness of Project SoL, purchasing patterns, overweight and obesity among children in high-intensity communities, and sensory education and food literacy of children. These summative sub-studies are described briefly below.

#### 3.2.1. Health Behaviours among Families in High-Intensity Communities 

Objective: To assess the effects of the SoL intervention on measures of behaviours and attitudes among families and their 3–8-year-old children in the three local, high-intensity communities.

Design: The data are collected in Bornholm and Odsherred at baseline, mid-term after one year and post-intervention after 19 months. The sampling is done at school level. The number of eligible children at baseline is a total of 443 children from the intervention communities and 418 from the control communities. However, the number of eligible children varies from year to year because of changes in the sample population caused by families’ in- and out-migration to or from communities or the child’s change of school or childcare centre.

Data collection methods: The Family Questionnaire (FQ) is distributed to the parents through the participating childcare centres and schools in which the intervention is delivered. The variables are the socio-demographic characteristics of the parents; self-reported weight and height of the children; self-reported health behaviours (children’s physical activity, cooking and shopping); parents’ attitudes to cooking and shopping; use of media; social capital. The questionnaires is mainly developed from the Danish National Health Profile Study [18] KidsActive-Q [19] and the Neighborhood Study [20]. Furthermore, a parent-administered semi-quantitative food-frequency questionnaire, referred to as Child Food Frequency Questionnaire (CFFQ), capturing children’s eating habits is also distributed to the parents. The CFFQ is validated in a sample of 53 three- to eight-year-old children, using four-day estimated food records as a reference. Reproducibility (*n* = 48) is measured by repeated FFQ administrations four weeks apart. For most food items, a moderate level of relative validity and reproducibility is observed for the assessment of food intake [21]. The CFFQ has a recall period of 4 weeks and includes twenty-two questions covering 183 food items. The CFFQ assesses the consumption of food groups related to the intervention’s target behaviours: intake of vegetables (total and coarse), fruits, fish, whole grains, candy (also known as sweets and chocolates) and sweet drinks. The CFFQ also contains information on the child’s date of birth, sex, and information of the reporter (mother, father, other).

Data analysis: We use a longitudinal linear mixed model (SAS PROC MIXED) to assess differences in dietary outcomes between the intervention and the control group. An unstructured residual covariance matrix is used to account for serial correlation of observations from the same individual over time. To account for similarities among children within the same community and school or childcare centre, the model includes two random effects, local community and school or childcare centre. We include group (intervention/control) and visit (baseline/first follow-up/final follow-up) and the interaction between group and visit as fixed effects. The model also adjusts for relevant covariates e.g., sex, age, education, household income and family type depending of the selected outcome variable.

#### 3.2.2. Health Behaviours among Citizens at Municipality Level

Objective: To assess the effect of the SoL intervention on the health behaviour among citizens in the municipal population, with special focus on the effect of the mass media intervention.

Design: A self-administrated questionnaire is mailed to a total of about 1500 citizens, randomly selected based on CPR address extraction, in both two municipalities, Bornholm and Odsherred, at baseline, mid-term (after one year) and post-intervention (after 19 months).

Data collection methods: The Citizen Questionnaire (CQ) used mainly selects questions from The National Health Profile questionnaire [18], including data on socio-demographic characteristics, dietary intake, physical intake, smoking, alcohol, health status and self-rated health. There is a particular focus on dietary behaviours such as the frequencies of fruit, vegetables, whole grain and fish intake and on the location of shopping opportunities and shopping frequencies. In addition, the questionnaire aims at mapping eating practices, which including who does the cooking, how long time is spent, who and what decides the menu. Finally, there are questions on motivation for changing lifestyle, perceived barriers for changing of lifestyle, self-reported health and lifestyle, well-being, awareness of health, weight, height as well as social capital. Data analysis: See Section 3.2.1 above.

#### 3.2.3. Awareness of Project SoL

Objective: To assess the effect on awareness of the mass media intervention at Bornholm and of the multicomponent intervention within the three local communities.

Design: Unlike the other parts of the intervention components of Project SoL that is delivered in the high intensity areas, the mass media part of the intervention is broadcasted to the whole of the Bornholm via TV, radio, newspaper and social media (Facebook). As such, the sampling unit is the community. The quasi-experimental sub-study comprised of: (1) a comparison of the awareness of Project SoL among citizens at Bornholm and Odsherred at baseline, 1 year and 2 years follow-up; (2) pre- and post-consumer interviews within the three high-intensity intervention communities 

Data collection methods: The quantitative data on media use and awareness of the SoL project are collected through the CQ questionnaire mentioned above (Section 3.2.2), as well as telephone- (*n* = 416) and short customer interviews (*n* = 347). The data on citizen media awareness and media habits are collected through telephone awareness interviews with citizens in each of the respective areas. Furthermore, short customer awareness interviews are carried out with a sample of consumers in the involved food stores in the three high-intensity intervention communities of Bornholm at baseline (*n* = 186) and 2 years follow-up (*n* = 161 citizens).

Data analysis: The data from questionnaires, telephone- and short customer interviews are analyzed in the statistical software SAS 9.4 (SAS Institute, Inc., Cary, NC, USA).

#### 3.2.4. Purchasing Patterns

Objective: To assess the effect of the multi-component intervention including the mass media intervention and specific supermarket interventions on consumers’ food purchase patterns.

Design: All the main supermarkets on Bornholm and in Odsherred (40 supermarkets) provides sales data apart from one small retailer on Bornholm that declines to participate in the study. The evaluation focuses on sales of fruits, vegetables, whole grains, fish, sugary beverages, unhealthy snacks, candy and cakes [22,23]. For a complementary qualitative study, see Section 4.2.2.

Data collection methods: Weekly data on sales volumes for all products sold before and during the intervention period were collected from the headquarters of the supermarket chains.

Data analysis: A longitudinal linear mixed-effects model (SAS PROC MIXED) with the logged index numbers regressed on a time dependent intervention variable is fitted to evaluate the effect of the intervention on food sales. We use a random effect for supermarket to allow for heterogeneity among supermarkets, and an autoregressive AR1 correlation structure to account for larger similarities of observations closer in time on the same supermarket.

#### 3.2.5. Overweight and Obesity among Children in High-Intensity Communities

Objective: To determine whether the high-intensity SoL intervention had an effect on prevention of overweight in three- to eight-year-old children in the three high-intensity communities.

Design: All children in the participating childcare centres and primary schools are invited to participate in the anthropometric measurements at baseline and at follow-up. The number of eligible children at baseline is 443 children from the intervention communities and 418 from the control communities [24].

Data collection methods: The height, weight and waist circumference of participating children is measured with the participants being barefooted and in light clothing. This took place in the involved childcare centres and primary schools during normal work hours. Height is measured to the nearest 0.1 cm using a portable stadiometer (Leicester Height Measure, Child Growth Foundation, Chiswick, UK) and body weight is measured to the nearest 0.1 kg using a digital scale (Tanita BWB-800, Tanita Corp., Arlington Heights, IL, USA). Waist circumference is measured to the nearest 0.5 cm midway between the lowest rib and iliac crest. Waist circumference is measured in triplicate.

Data analysis: BMI is calculated as weight in kilograms divided by the height in meters squared and converted to Z-scores using the LMS method (skewness-L, mean-M and coefficient of variation-S) with the use of Danish reference data [25]. The BMI Z-scores provided an age- and gender-standardized measure of overweight and obesity. We use a longitudinal linear mixed model to assess differences in anthropometric outcomes between the intervention and control groups (See Section 3.2.1 above).

#### 3.2.6. Sensory Education and Food Literacy of Children

Objective: To examine the effect of taste and sensory education workshops on familiarity, liking and intake of fruits and vegetables among children in childcare centres and schools.

Design: Sensory education workshops are held in childcare centres and primary schools including play, cooking and exposure to fruit and vegetables. The participants are children and professionals of the institutions. The effect variables of the sensory education are inspired by the Sapere method [26]. The method is developed to train young people sensory aspects of food and eating and has been successfully tested in both kindergarten and school settings in a number of countries. The Sapere components are evaluated using a quasi-experimental design comparing the intervention with the control municipality at baseline and post intervention follow-up. The sample size including childcare centres and schools is (*n* = 405) children in the intervention group and (*n* = 262) in the control group at baseline.

Data collection methods: Knowledge about common fruits and vegetables available in Denmark (12 different fruits/vegetables in schools, eight in childcare centres) are measured at baseline and post intervention. A game of bingo is used to assess the children’s ability to identify the different fruits and vegetables using colour codes. Furthermore, liking of four selected vegetables/fruits (parsnip, apple, beet root and Brussels sprouts) is measured using taste samples and the smiley scale (7-points in schools, 5-points in childcare centres) at baseline and post intervention. Finally, an exposure study is conducted to investigate the development in acceptance and intake of Brussels sprouts.

Data analysis: The data on the familiarity with fruits and vegetables (number of correct out of respectively 8 and 12 possible) are analysed using non-parametric tests to compare baseline with post intervention. Similarly, the liking is analysed in the statistical software SAS 9.4 (SAS Institute, Inc., Cary, NC, USA) to compare pre-post intervention differences.

## 4. Formative Research

### 4.1. General Issues

The formative evaluation of Project SoL takes the programmed theory [11] as its conceptual inspiration. We select a number of CMO configurations for in-depth studies as will be described below. The selection is based on an assessment of where the most challenging mechanisms in relation to behavioural change would be. Thus, specific studies are conducted on children’s perceptions of health, supermarket retailers’ perceptions and practices, media stakeholders’ perceptions on their involvement in Project SoL as well as the perspectives of the members of the Local Action Groups.

### 4.2. The Formative Studies

#### 4.2.1. Children’s Perspectives on Health

Objective: To learn about children’s perceptions and visions for a socially and physically healthier school environment with the view to initiate changes in their local institutions and surroundings.

Research design: This study is based on action research and involved one of the three primary schools targeted by the project at Bornholm. The study is organized in three phases—a conceptual phase, a practical phase and a follow-up phase. In the conceptual phase, about 50 children from two 2nd grade classes participate in a future workshop of a week’s duration. The school and classes are representative for high-intensity communities and are sampled based on their high level of motivation to participate. The methodology built on the work of Jungk and Müllert [27] to stimulate children to express their perceptions about health and illustrate their visions for a healthier school environment through drawings, collages and models. In the practical phase, about 250 school children from 0–3rd grade are engaged in social and physical activities for improving the school environment involving eight workshops addressing different themes (e.g., role plays, aesthetic painting, nature fitness and preparing healthy meals) that are developed from the outcomes of the conceptual phase. In the follow-up phase, more senior pupils, school board and school management team members are involved in discussions and planning of more long-term structural improvements originating from the expressed needs of the children.

Data collection methods: In the conceptual phase, the researchers organize and facilitate participatory processes for the children based on the future creation-workshop approach as well as focus group discussions. In the practical phase, the researchers make structured participant and non-participant observations about the engagement and actions of children, teachers and relatives in the workshops. In the follow-up phase, the researchers make structured participant observations at meetings in arenas defined by adults including teachers meetings and meetings in governing bodies at the school. This is supplemented by minutes from the meetings and by e-mail correspondence between local stakeholders and researchers on the developments.

Data analysis: Qualitative content analysis involving coding and categorizing meaning units into sub-themes related to children’s health perceptions and visionary aspects of the social and physical school environment. Interview data are analyzed with reference to social learning theory [28] and action research theory [29].

#### 4.2.2. Supermarket Retailers’ Perceptions and Practices

Objective: To examine how food retailers perceived the feasibility of integrating health promotion aspects in their daily work practices, and how they participate in Project SoL in practice.

Design: Exploratory study using a practice-theoretical approach and qualitative methods. Local supermarket staff and managers as well as national chain managers and directors from the participating food retail groups are purposively sampled based on the principle of maximum variance and interviewed at baseline and follow-up utilising a question guide.

Data collection methods: The study includes 27 semi-structured interviews. Moreover, data includes minutes and audio recordings collected throughout the project from development, planning and evaluation meetings. Furthermore, implementation reports and log book notes are made at store visits.

Data analysis: Analysis of perceptions and practices based on interview and observation data, including drivers and barriers for engaging in health-promoting practices in the light of prevailing business practices. The steps of a theoretical thematic analysis [30] are followed in the coding and theme-making across data within a conceptual framework drawing primarily on practice theory [31].

#### 4.2.3. Media Stakeholders’ Perceptions

Objective: To investigate the role of the mass media stakeholders including the local television, radio and newspaper in health promotion in the context of Project SoL.

Design: Representatives from the local media at Bornholm and in Odsherred are invited for an interview regarding their view on the SoL-project. The informants were six purposively selected mass media stakeholders, three at Bornholm (TV2 Bornholm, Bornholm’s Gazette, Radio: DR P4 Bornholm), and three at Odsherred (TV2 East). The weekly paper of Odsherred, DR P4 Zealand) is invited in order to ensure a maximum variety of key players among the local media.

Data collection methods: Semi-structured interviews are conducted based on an interview guide.

Data analysis: Recordings of the semi-structured interviews are transcribed verbatim, coded and analyzed using the qualitative data analysis software NVivo (Nvivo 10, QSR International Pty Ltd., 1999–2013). A theoretical thematic analysis using a latent approach [30] is used to describe the content, and find repeated patterns of meaning that were valid across mass media stakeholders thereby exploring the role of mass media in health promotion.

#### 4.2.4. Local Action Group Members’ Perceptions

Objective: To explore the perceived motivations and barriers of community stakeholders and citizens to contribute to and take responsibility for social and health promotion action in the local high-intensity communities, with the view to promote long-term anchoring and sustainability of the interventions.

Design: The study is carried out in three phases: (1) establishment of Local Action Groups (LAGs); (2) assessment of motivations and barriers for participation; and (3) follow-up assessment of the sustainability of the actions taken. In the first phase, a LAG is established and supported administratively by researchers in each of the three high-intensity intervention communities on Bornholm. Researchers attended the LAG meetings and supported their organization and implementation of community activities. In the second phase, which is implemented about one year after establishment of the LAGs, members sampled purposively to ensure maximum variance are interviewed about their experiences and perceptions of involvement and participation. In the third phase, the degree to which the LAGs were integrated into local community structures and remained active after project completion is assessed.

Data collection methods: During the first phase, data are collected through structured participant observations, informal discussions, minutes from group meetings and email correspondence with stakeholders on developments. During the second phase, data were obtained from semi-structured in-depth interviews with members of the LAGs. During the third phase, data were obtained from non-participant observations, informal stakeholder conversations and semi-structured interviews.

Data analysis: Interview data were analyzed with reference to self-determination theory [32]. This is done using qualitative content analysis involving coding and categorizing of meaning units into sub-themes related to motivations and barriers as perceived by members of the LAGs.

## 5. Discussion

The present paper provides an outline of Project SoL’s research and evaluation design. Inspired by realistic evaluation [11], a programmed theory is developed and key CMOs are identified on which summative and formative studies are conducted. The evaluation of Project SoL includes measurements of a range of behaviour changes within the target groups as proxies for health outcomes. This constitutes the summative evaluation of the project together with anthropometrics measurements among children, knowledge and preferences of fruit and vegetables and awareness of Project SoL. In addition, the evaluation includes efforts to understand the perceptions of key stakeholders such as children, staff in supermarkets and media stakeholders, as well as a description of the complex social processes taking place in community systems undergoing change in relation to the LAGs. These sub-studies constitutes the formative part of the evaluation.

There are a number of lessons learnt associated with the implementation of a research-based development project such as Project SoL. These are outlined below in separate though partly overlapping themes. These themes reflect the researchers’ perspectives as no systematic effort are made to capture the practitioners’ perspectives.

### 5.1. Lessons Learnt

#### 5.1.1. Evaluating Complex, Multi-Component Interventions 

Complex, multi-component interventions that are implemented in multiple settings and have strong participatory components require compatible scientific approaches to evaluation of outcomes [13,14] and processes. However, as pointed out by Thoreogood & Coombes [33] the trend in health promotion is to emphasize outcome and impact of interventions while giving less attention to process and context [33,34], thereby missing the opportunity of gaining important insights into what worked, why, how and for whom. The challenges have been summarized by [13] as: (1) standardizing the design and delivering the interventions in a way that is sensitive to the features of the local context; (2) the organizational and logistical difficulty of applying experimental methods to service or policy change; and (3) the length and complexity of the causal chains linking intervention with outcome. Furthermore, it is rarely possible to measure how much each intervention component affects the target group. Instead complex interventions focus on the totality of the different intervention components and actions as compared to non-intervention sites. Furthermore, the process of developing and evaluating a complex intervention includes several phases, but that they may not follow a linear sequence, and experimental designs are to be preferred to observational designs in most circumstances, but that such designs may not always be practicable.

Project SoL combined the participatory approach to health promotion with the ambition to apply a rather rigorous summative evaluation [9]. This is handled by combining the strengths of the controlled, pre/post quasi-experimental design for summative sub-studies with complementary qualitative formative sub-studies. Project SoL addresses this issue by using a realistic evaluation approach. Based on a programmed theory, the research focuses on specific and manageable CMO configurations that can be evaluated in detail, thereby allowing us to distinguish between errors of implementation and/or theory.

In retrospect it is clear that the magnitude of the evaluation task, has not been fully clear to the research group from the start. It takes major efforts to conduct and coordinate the various summative and formative sub-studies. More emphasis and detailed planning can recommend to be done up front in order to better align tasks and resources. We find that the realistic evaluation and mixed methods approaches are useful tools and concepts to guide our research.

#### 5.1.2. Practicing Cross-Disciplinary Research

The advantages of applying cross-disciplinary research principles are well known within public health research as a means of finding research-based solutions for practical problems as well as identifying innovative new research questions. At the same time the cross-disciplinary teams need to overcome challenges by harmonizing issues of research design and data collection methods, as well as more profound disparities of paradigms and power relations [35].

Project SoL brings together different types of public health expertise such as public health, epidemiology, health promotion, integrated food studies, sensory and consumer sciences, educational and social sciences. Cross-disciplinarily is seen as an important prerequisite for the project team to develop the complex intervention and research design. The wide diversity of traditions among disciplines leads to heated debates about research design and implementation processes in the preparatory stages of the project, but compromises are gradually found whereby research goals and synergies can be achieved. This necessary and trying process consumes a significant amount of time and energy. Cross-disciplinary collaboration is resource demanding in terms of labour resources, time consumption and operating costs. The project team approaches this challenge by allocating a significant amount of time to meetings and scientific discussions and reflections during the projects.

#### 5.1.3. Collaboration between Researchers and Community Practitioners

A close and equitable collaboration with local non-academic stakeholders is a precondition for the implementation of an intervention such as Project SoL [9]. These partners include families, managers and staff members in supermarkets and in local institutions, and representatives from civil society and the public administration at municipality level. There is a potential tension between conditions and relatively short timelines of practical work and an ambitious evaluation agenda rooted in the researchers attempt to create a solid evidence base for possible public health actions. Studies on development and implementation of community health interventions point to the risk of research protocols being too much driven by the researchers’ needs for consistent protocols and the ambition of showing measurable quantitative effects rather than by the needs of the end-users [36].

Over time, close collaborative ties developed between the researchers and the various stakeholders. As with the internal interaction between the various disciplines, it is time-consuming and not always easy, but it is an essential part of the process to allocate the necessary time. The main lesson learnt here is that a much more systematic and focused effort should be made at the very beginning of the project, an approach that we subsequently establish as a rule in other similar projects.

#### 5.1.4. Striking the Internal Balance between Research and Implementation

Although based on an idea originating from the local media stakeholder TV2 Bornholm, Project SoL is initiated by the researchers. Consequently, they play a leading role in the mobilization of local stakeholders and implementation of many of the interventions especially during the first year. The researchers are very aware of challenges in terms of potential unclear roles and insufficient time allocated to the research which added to the strain on the work load during the first part of the project. A separate research forum for researchers and students of the project established allows for academic discussion, interactional and coordination at the scientific level. The research forum links to the coordinating body of the project through senior researchers from the three partner organizations and the local project coordinator. This secures proper and timely dialogue between the project’s research component and its intervention component. This dialogue is intense constantly during the entire project period because action research is practiced as a key methodology approach in most of the project activities.

### 5.2. Building Research Capacity

Project SoL makes a significant contribution to research capacity building by including not only PhD students and postdoc researchers, but in addition a large number of pre- and postgraduate students whose projects supplemented the main research activities. The inclusion of pre- and postgraduate students from a range of different universities and disciplines is at the same time contributing to the cross-disciplinarily of the project since it includes a multitude of different research disciplines such as public health, epidemiology, integrated food studies, health pedagogy, sensory and consumer sciences as well as social science. Though the directions of such spin-off and add-on studies are somewhat unpredictable due to their ‘university autonomy’, they are found to be contributing to the main project in unexpected ways. Thus, a win-win situation is created in which these associated studies served to create new inspiration to Project SoL and at the same time serves as a field of research education for future generations of experts within the field of community-based health promotion research. It is our experience that pre- and post-graduate students can contribute significantly when supervised sufficiently.

### 5.3. Implications for Research

Project SoL adds to the growing knowledge in the field of complex, community-based health promotion interventions [37,38]. These studies point out some important challenges related to developing and evaluating complex community-based interventions. Overall, there is a need to document many more cases of community-based intervention projects combining complex interventions and ambitious research components within health promotion as well as within other sectors.

## 6. Conclusions

Project SoL adds to the growing knowledge in the field of combined implementation and evaluation of complex community-based health promotion interventions. The present article outlines the research design including summative and formative elements based on a programmed theory of realistic evaluation. Seen together with the complementary article of [9] on the planning and implementation of the health promotion intervention, it is the hope that it may guide new groups of professionals in their endeavours.

## Figures and Tables

**Table 1:** Overview of formative and summative data collecting methods.

DCM/Questionnaire	Abbreviation	Respondents	Venue	Time			Article Section
				Pre	Intermediate	Post	
Citizen questionnaire	CQ	Citizens	Bornholm & Odsherred	X	X	X	3.1.1.2
Family questionnaire	FQ	Parents	Bornholm & Odsherred	X		X	3.1.1.2
Child food frequency questionnaire	CFFQ	Parents	Bornholm & Odsherred	X	X	X	3.1.1.2
Phone interviews		Citizens	Bornholm & Odsherred	X (*n* = 70&70)	X (*n* = 70&70)	X (*n* = 70&70)	3.1.2
Short customer interviews		Customers	Bornholm & Odsherred				3.1.2
Purchase		Customers	Bornholm & Odsherred	Weekly			3.1.3
Anthropometrics		Children	Bornholm & Odsherred	X (*n* = 443)		X (*n* = 418)	3.1.4

## Abbreviations

CG	Citizen Questionnaire
CFFQ	Child Food Frequency Questionnaire
CMO	Nodes of Context, Mechanism, Outcome as part of realistic evaluation
FQ	Family Questionnaire
LAG	Local Action Group
SoL	Health and Local Community—from the Danish ‘Sundhed og Lokalsamfund’.

## Appendix A

**Table A1:** List of other associated SoL research projects not mentioned in the main text.

Title	Authors, University, Year	Level	Purpose
Healthy business or business as usual? A practice-oriented perspective on a supermarket-based health promotion intervention	Winkler LL. Research Centre for Prevention and Health (RCPH), Glostrup, Denmark, 2017.	PhD thesis	To examine the supermarket as a setting for health promotion from an everyday perspective primarily based on practice theory.
Effects of a multi-component community-based health promotion intervention on eating behavior and overweight in children. Results from the Health and Local community (HLC) project	Tine Buch-Andersen. 2012–2016.	PhD thesis	The overall aim of the present PhD project was to examine the effects of the HLC intervention on eating behavior and overweight of 3–8-year-old children.
Assessing Action and Intervention Possibilities in Local Communities—the Local Community Foodscape Assessment Tool (LC-FAT)	Jørgensen, N.K., Bundgård L.M.I., Aalborg University, Copenhagen, Integrated Food Studies. 2015.	Master’s thesis	
Differences between policies and practice. A study of the government’s, the munnicipality’ and the daycare center’s health policies in addition to nursery workers’ and children’s perspectives on health-related practices	Hyllekilde Andersen, L.;), University of Southern Denmark, Faculty of Health science. 2015.	Master’s thesis	To contribute with knowledge about how health policies and health practices participate in shaping the environment in which the children in daycare center (case SpilOp’en) act every day.
The influence of the SoL project on health behaviour of child families at Bornholm.	Pedersen MB. Faculty of Health Sciences, University of Southern Denmark, 2014.	Master’s thesis	To get a thorough understanding of how Projekt SoL affects the capability to live healthily of the families with young children.
A qualitative study of participation in a community health promotion initiative—from a process and participant perspective.	Sørensen MESB. Department of Psychology and Educational Studies, Department of Environmental, Social and Spatial Change University of Roskilde, 2014.	Master’s thesis	To describe the elements that enable and challenge the participation of local people in a health promotion project.
Implementation of school meal programmes and promotion of healthy eating habits among pupils—A case study	Heerup, C.T; Bødker, S., Aalborg University, Copenhagen, Integrated Food Studies. 2014.	Master’s thesis	To examine (1) how a school meal programme (Paradisbakkeskolen) can contribute to promote healthy eating habits among pupils, (2) how the new school legislation could be used to rethink food and meals in schools.
Project SoL’s impact upon the health behaviour among families with young children on Bornholm	Brogaard Pedersen, M., University of Southern Denmark, Faculty of Health science. 2014.	Master’s thesis	To get a thourough understanding of how Project SoL affects the capability of families with young children to live healthy lives.
Children’s perspectives on health—and their participation in health promoting activities through future creating workshops	Schmidt C. Department of Public Health, University of Southern, 2013.	Master’s thesis	To explore children’s viewpoints on health and to discuss if and how children can perform as participants in future workshops
Barriers and possibilities for teachers’ implementation of a health promotion project at a Danish primary school. A process evaluation of the implementation of the SoL project	Christensen KH, Landbo MVE. Institute of Public Health, University of Copenhagen, 2013.	Master’s thesis	To describe the barriers and opportunities for front staff in a primary school to experince ownership of a local intervention and the significance of these barriers and opportunities for implementing health promotion activities.
An investigation of the local food system on Bornholm: a case study on school meal procurement policies	Brandt Jensen, C., Aalborg University, Copenhagen, Integrated Food Studies. 2013.	Master’s thesis	To investigate (1) the local food system at Bornholm and (2) how local food is being integrated in Bornholm. Use of mixed methods, (literature review, actor-mapping, interviews).
